# Developing Connectedness to Nature in Urban Outdoor Settings: A Potential Pathway Through Awe, Solitude, and Leisure

**DOI:** 10.3389/fpsyg.2022.940939

**Published:** 2022-07-11

**Authors:** Timothy J. Mateer

**Affiliations:** Department of Recreation, Park, and Tourism Management, The Pennsylvania State University, University Park, PA, United States

**Keywords:** solitude, urban parks, awe, connectedness to nature, leisure

## Abstract

Outdoor leisure experiences may represent an understudied yet effective pathway to promote connectedness to nature for urban park visitors. In contrast to outdoor recreation, this critical essay argues outdoor leisure more heavily emphasizes eudaimonic sentiments and intrinsic motivation in comparison with the goal-oriented and hedonic nature of outdoor recreation. It is further argued that two specific social psychological constructs, awe and solitude, may be especially useful in promoting leisure experiences in urban outdoor spaces. Relevant philosophical and social psychological literature is reviewed and synthesized to outline how land managers and environmental educators may facilitate experiences of awe and solitude to better promote contexts for experiencing outdoor leisure in urban parks. Specifically, reviewed literature suggests that utilizing the recreation opportunity spectrum framework and co-creative processes may be an effective path forward in better supporting urban park environments that are conducive to awe, solitude, and leisure. The review and synthesis of this research may ultimately guide environmental educators, land managers, and researchers in ways to more effectively support connectedness to nature *via* outdoor leisure experiences as an outcome for visitors to outdoor urban spaces.

## Introduction

Currently, over 55% of the world’s population resides in urban areas (World Health Organization, 2021). While urban centers are often cited as providing an array of social and cultural benefits for residents (e.g., Clark and Kahn, 1988; Machado et al., 2013; Borgoni et al., 2018), a range of psychological (Nisbet and Zelenski, 2011), infrastructural (Biernacka and Kronenberg, 2018), and sociocultural (Rigolon, 2017; Mowatt, 2018) barriers may result in urban residents feeling disconnected from the natural world. This disconnect may be concerning regarding the well-being of social-ecological systems (i.e., both humans and more-than-human nature), as connection and access to nature links to numerous individual and collective health benefits for humans (e.g., lower levels of anxiety, higher levels of prosocial emotions; Kuo, 2015; Jennings et al., 2017; Lopes et al., 2020; McConnell and Jacobs, 2020) and urban ecological systems (e.g., Anderson and Minor, 2017). To bridge this physical and psychological gap between urban residents and outdoor spaces, practitioners often use outdoor recreation as a pathway to connect individuals with the outdoors (e.g., Thompson et al., 2005; Wolch et al., 2011). Outdoor recreation broadly refers to an activity occurring during one’s free time that involves participants interacting with the natural world in some manner (Jenkins and Pigram, 2003; Lackey et al., 2021). Although practitioners often uncritically accept outdoor recreation as an effective tool in developing a relationship between humans and outdoor spaces in urban areas (e.g., Outdoor Foundation, 2020), some limitations may exist in relying too heavily on outdoor recreation, given such pursuits often emphasize hedonic well-being and extrinsically motivated, goal-oriented behaviors (Holba, 2013; Dattilo and Lopez Frias, 2020). Even though these pursuits can have beneficial outcomes for outdoor recreation participants, finding ways to also promote eudaimonic well-being (Ryff and Singer, 2008; Huta and Waterman, 2014) and intrinsic motivation (Ryan and Deci, 2000) may provide alternative beneficial outcomes for individuals in ways that complement those encouraged by outdoor recreation.

In contrast to outdoor recreation, outdoor *leisure* may provide this alternative pathway to connect urban residents with outdoor spaces. While recreation and leisure are often used interchangeably, some scholars assert that the terms have different historical origins as well as practical connotations (e.g., Holba, 2013; Dattilo and Lopez Frias, 2020). For instance, Holba (2013) argues that leisure represents an action that holistically consumes an individual’s mental state, arising from intrinsic motivation and thoughtfulness when participating in the chosen activity, and contrasts leisure and recreation by stating, “The most obvious difference between transformative leisure and recreation is the action of contemplation—transformative leisure has it and recreation does not” (p: 22). Such contemplation (i.e., leisure) without a specific purpose is believed to be essential to the human condition (Pieper, 1963). Dattilo and Lopez Frias (2020) align with Holba’s (2013) assertion, stating that moments of leisure may occur during recreation, but engagement in recreation activities does not constitute a leisure experience in and of itself.

In further contrasting outdoor recreation and outdoor leisure, as alluded to previously, the former primarily promotes hedonic well-being while the latter emphasizes eudaimonic well-being. Promoting eudaimonic experiences *via* outdoor leisure may help develop an authentic and personal relationship between urban residents and the natural world in a manner that is not emphasized in the hedonic nature of outdoor recreation. In keeping with the broader approach taken throughout this critical essay, eudaimonia and hedonia are utilized in a manner that integrates both philosophical and social psychological perspectives on the terms (e.g., Deci and Ryan, 2008; Ryff and Singer, 2008; Huta and Waterman, 2014). The conceptual distinction between hedonia and eudaimonia can be traced to Aristotle’s (2004/ca. 350 B.C.E.) discussion on the nature of happiness and well-being, and interest regarding the terms in a social psychological sense can be traced to Ryan and Deci’s (2001) prominent literature review. Aristotle (2004/ca. 350 B.C.E.) asserts that happiness exists as the primary objective of life, but individuals differ on what constitutes the nature of this happiness (i.e., eudaimonic versus hedonic conceptualizations). Hedonic well-being largely aligns with what Aristotle (2004/ca. 350 B.C.E.) describes as the pursuit of pleasant and material-based well-being, a path toward what he acknowledges would be an enjoyable life, though potentially not as deep-seeded with meaning as eudaimonia. Social psychologists have built upon this philosophical conceptualization to describe hedonic well-being as the presence of pleasure and the avoidance of negative affect (e.g., Lengieza et al., 2019). Recreation’s goal-oriented nature often prioritizes the pursuit of such hedonic objectives. In contrast, as summarized by Aristotle (2004/ca. 350 B.C.E.) and Ryff and Singer (2008) asserts that eudaimonia is supported by pursuing a virtuous life, one that strives for balance between excess and deficiency. Through contemplation and striving for this balance, an individual may find a way forward in life that allows them to actualize their true nature [i.e., pursuing an intrinsically inspired path; Aristotle (2004/ca. 350 B.C.E.)]. As it is relevant to leisure experiences, many social psychologists have expanded Aristotle’s (2004/ca. 350 B.C.E.) original conceptualization of eudaimonia to describe human well-being in a manner that balances several complementary dimensions including: self-reflection, personal meaning, authenticity, and intrinsic motivation (Ryan and Deci, 2001; Ryff and Singer, 2008; Huta and Waterman, 2014; Lengieza et al., 2019). Scholars have asserted that leisure, in contrast to recreation, may provide space to pursue these ideals (e.g., Holba, 2013; Dattilo and Lopez Frias, 2020). Regarding outdoor leisure in urban outdoor spaces, eudaimonic experiences in the outdoors may support connectedness to nature in a manner that is personal, authentic, and intrinsically motivated.

Two key components may be especially useful in facilitating outdoor leisure experiences: awe and solitude. Awe broadly refers to a transcendental feeling facilitated by being in the presence of something vast (Bai et al., 2017). Alternatively, solitude is generally characterized by self-reflective thoughts and feelings facilitated by being alone (Long et al., 2003). Each of these components, discussed in greater length further in this critical essay, may allow for the outdoor environment to facilitate intrinsic and contemplative moments inherent in the eudaimonic nature of leisure experiences (Holba, 2013; Dattilo and Lopez Frias, 2020). Given the potential benefits associated with connecting urban residents to outdoor spaces, environmental educators and land managers may look to experiences of awe and solitude as mechanisms to promote outdoor leisure opportunities that complement outdoor recreation. In turn, these contextual factors may enhance both social and environmental health outcomes by developing a meaningful connection between urban residents and the natural world (Kuo, 2015; Jennings et al., 2017).

This critical essay intends to provide a framework for land managers, educators, and academics to facilitate contexts supportive of outdoor leisure for urban residents. Specifically, this writing has three primary purposes: (a) to explore the philosophical and psychological basis of awe and solitude facilitating outdoor leisure experiences, (b) to review current academic literature on what is known about awe, solitude, and leisure in urban outdoor spaces specifically, (c) and to provide guidance for land managers and environmental educators on how to facilitate these experiences. As done thus far, the terms “natural world,” “nature,” and “outdoor spaces” are used interchangeably throughout this writing. These terms align with the thinking of scholars across cultures (e.g., Asian, Indigenous American, Euro-American) that such terms encapsulate ecological systems that are dynamic over space and time and include living beings embedded within these systems (Leopold, 1949; Talukder, 2014; Kimmerer, 2015). Connection to nature, the outcome of outdoor leisure experiences explored in this paper, is defined by Lengieza and Swim (2021), referring to the “psychological joining of nature and the self which manifests as a sense of oneness with nature” (p: 2). In addition, it should be noted that the statements presented here primarily center within a Euro-American academic context within which the author is based.

## Leisure in the Outdoor Context

The following section outlines ways awe and solitude may support outdoor leisure experiences. How outdoor leisure may promote connectedness to nature is also explored. This, in turn, provides the basis for the second section of this critical essay that explores how such constructs have been understood in urban outdoor spaces specifically.

### Awe and the Outdoor Leisure Experience

If leisure in the outdoor context is contemplative, intrinsically motivated, and mindful (aligning with a eudaimonic perspective on well-being), awe may play a role in how outdoor leisure diverges from outdoor recreation. Awe can be conceptualized through the atmospheric lens as described by German philosopher Hermann Schmitz (Kazig, 2016). From this perspective, emotion is not bounded by the bodily self. Rather, emotion flows outward and can be influenced by contextual factors within which it is embedded (Kazig, 2016). Regarding awe specifically, Bai et al. (2017) assert that awe is “defined by two central appraisals: that one is in the presence of something vast, and that the elicitor transcends one’s current frame of reference for understanding the world” (p: 186). Furthermore, McShane (2018) expands this conceptualization by stating that awe has an outward-facing element to it. In other words, someone is normally “in awe” of an external object or phenomenon such as mountains, a hurricane, or innumerable other focal points (McShane, 2018). Although the outward-facing nature of awe may seem contradictory to the intrinsic nature of leisure (e.g., Holba, 2013), awe is a reflexive feeling. Although awe partially directs attention externally, the root of the appraisal ultimately returns to how individuals perceive a diminished sense of self in relation to their broader surroundings (Bai et al., 2017). Research in the field of social psychology further builds upon this conception of awe in the outdoors; for example, Bethelmy and Corraliza (2019) assert that awe consists of five elements: fear, threat, vulnerability, fragility, and respect for nature. Losing oneself in the grandeur of the natural environment closely parallels what Pieper (1963) defines as a philosophical act. Such philosophizing, a central element to experiencing leisure, allows humans “to go beyond the trusted enclosures of the normal, customary day-to-day reality of the whole of existing things, to go beyond the ‘environment’ to the ‘world’ in which that environment is enclosed” (Pieper, 1963, p: 111). Further, eudaimonia, and concurrent moments of leisure, may be supported by the contemplation that is spurred by experiences of awe (e.g., Graves et al., 2020).

Experiencing awe and leisure in relation to urban outdoor spaces may specifically help individuals contemplate and gain perspective on their role in the broader social-ecological systems within which they exist (Bai et al., 2017; Bethelmy and Corraliza, 2019). If the eudaimonic nature of outdoor leisure supports authenticity and personal reflection, these direct, emotional experiences in the outdoors may play an important role in helping individuals develop a meaningful connection with the outdoors (Chawla, 1998; Heberlein, 2012; Williams and Chawla, 2016). Specifically, the intense and overwhelming emotions associated with awe may encourage individuals to conclude the natural world holds value beyond its economic and utilitarian value. For example, Leopold (1949) advocates for the intrinsic worth of ecosystems broadly through his “Land Ethic” philosophy. In making his points, he regularly refers to moments of awe he feels toward the natural environment. It is directly from these moments of intangible emotion that he derives many of his arguments. He writes:

Sometimes in June, when I see unearned dividends of dew hung on every lupine, I have doubts about the real poverty of the sands. On solvent farmlands lupines do not even grow, much less collect a daily rainbow of jewels. If they did, the weed-control officer, who seldom sees a dewy dawn, would doubtless insist that they be cut. Do economists know about lupines? (Leopold, 1949, p: 102).

Leopold (1949) contrasts the early morning beauty of wildflowers with the constant push for greater economic return in the United States, questioning what is lost when taking the latter approach. Scholars outside of the Euro-American context (e.g., Talukder, 2014; Kimmerer, 2015) have also shared similar conceptualizations of awe toward the natural world. Given awe and contemplation through outdoor leisure may lead to a diminished sense of self (Bai et al., 2017), such experiences invite individuals to contemplate where they fit into broader world systems.

### Solitude and the Outdoor Leisure Experience

In addition to feelings of awe, solitude may play a valuable role in maximizing individuals’ potential to experience leisure in urban outdoor areas. Contemplation plays a critical role in the eudaimonic nature of leisure (Holba, 2013), and solitude in outdoor settings may provide space for this contemplation. According to Long et al. (2003), solitude is a multi-faceted experience that, while alone, allows individuals to feel various positive emotions ranging from inner peace to creativity; solitude contrasts with loneliness which is commonly considered a negative emotion with individuals longing for contact with others. Moments of solitude in the outdoors may offer individuals the opportunity to escape from the “work-a-day world,” a key tenet of leisure experiences as defined by Pieper (1963). Managerial practices (Pilcher et al., 2009) and legislation (The Wilderness Act, 1964) in the United States institutionally support the independence and escape associated with solitude in the outdoors. For example, the Wilderness Act of 1964 stipulates that a wilderness in the United States is “recognized as an area where the earth and its community of life are untrammeled by man, where man himself is a visitor who does not remain” (p: 2). This intentional language codifies natural areas as a place to escape from the rush of daily life that is synonymous with existing in a capitalist society, a place to experience the outdoors in a personal manner that is integral to eudaimonia and leisure. The definition provided by the Wilderness Act of 1964 generally refers to large tracts of land separate from urban areas, indicating a level of tension on how to operationalize solitude in urban outdoor areas. The later sections of this critical essay further explore this tension.

Potentially due to this contrast with how many individuals live their daily lives, many people idealize solitude as an aspirational way of life. For example, individuals, such as Henry David Thoreau and his 2 years living alone along Walden Pond (Thoreau, 1948), have become canonized in Western culture for embracing solitude and the contemplative processes that can come with it. This builds upon Aristotle’s (2004/ca. 350 B.C.E.) previously established arguments that space for solitude, and subsequently contemplation, is necessary for living a virtuous life. According to some scholars (Leopold, 1949; Kimmerer, 2015), this virtuous way of life, which can be encouraged through leisure experiences, further requires individuals to behave in an ethical manner toward the natural world. Solitude in the outdoors provides the context for an escape from daily life, both physically and mentally, for individuals to contemplate and pursue this ideal (Pieper, 1963). Such contemplation further supports the eudaimonic nature of leisure in a manner that is not similarly encouraged by recreation and hedonia. The extensive research suggesting that exposure to natural sounds (as well as the absence of anthropogenic noise) enhances mood and attention (e.g., Benfield et al., 2014; Abbott et al., 2016), further bolsters the case that solitude may promote outdoor leisure. Research conducted in rural (e.g., Pilcher et al., 2009) and urban contexts (e.g., Gidlöf-Gunnarsson and Öhrström, 2007) support the value of natural sounds in this regard.

### Outdoor Leisure as a Context for Promoting Connectedness to Nature

Land managers and environmental educators in urban areas may be especially interested in awe and solitude as factors promoting outdoor leisure, given such experiences may help individuals develop a closer relationship with the natural environment. Previous scholars have articulated connectedness to nature in a variety of ways, with various philosophical threads asserting human consciousness, existence, and morality are inextricably linked to their embeddedness within the natural world (e.g., Leopold, 1949; Naess, 1973; Wilson, 1984; Kimmerer, 2015). For example, the “deep ecology” movement described by Naess (1973) argues that the natural environment holds intrinsic worth in parallel to the value frequently placed upon anthropocentric entities. Thus, humans and the natural world are linked by their intrinsic value (Naess, 1973). Alternatively, Wilson’s (1984) “biophilia” hypothesis asserts humans are innately attracted to other living things due to their shared evolutionary history. In parallel to these philosophical origins, social psychological research has explored how connectedness to nature ultimately influences human behavior. Psychological connectedness to nature has been linked to both human (e.g., Kuo, 2015; Lopes et al., 2020) and ecological (Nisbet et al., 2009) health. Regarding human well-being, a variety of individual and collective health benefits have been documented. Feeling psychologically close to nature is related to individuals holding stronger prosocial emotions (McConnell and Jacobs, 2020), enhanced ability to focus (Barbiero and Berto, 2018), and lower levels of anxiety (Martyn and Brymer, 2016). Regarding ecological well-being, connectedness to nature has been consistently linked to pro-environmental behavior in the environmental psychology literature (e.g., Mayer and Frantz, 2004; Nisbet et al., 2009). Thus, previous research indicates feeling a sense of psychological oneness with the natural world can support both human and ecological health.

As outdoor recreation generally focuses on participating in an activity to promote an intended outcome such as providing health benefits or filling free time (Jenkins and Pigram, 2003; Lackey et al., 2021), these activities can easily be co-opted for economic purposes or emphasize hedonic pleasure at the expense of eudaimonic sentiments (Simon and Alagona, 2013). The potentially utilitarian relationship with the natural environment promoted by outdoor recreation may not be enough to facilitate a meaningful relationship between humans and the remainder of the natural world. Leopold (1949) warns against this, stating, “We can be ethical only in relation to something we can see, feel, understand, love, or otherwise have faith in” (p: 214). If outdoor recreation is used to primarily serve instrumental outcomes, this emotional relationship with the land may be sacrificed at the expense of achieving these other goals. Notably, Høyem (2020) found reflection on human—nature relationships as a critical antecedent of outdoor recreationists adopting pro-environmental behaviors, suggesting the contemplative aspects of outdoor leisure may be effective in promoting a pro-environmental mindset for individuals.

**Figure 1:**
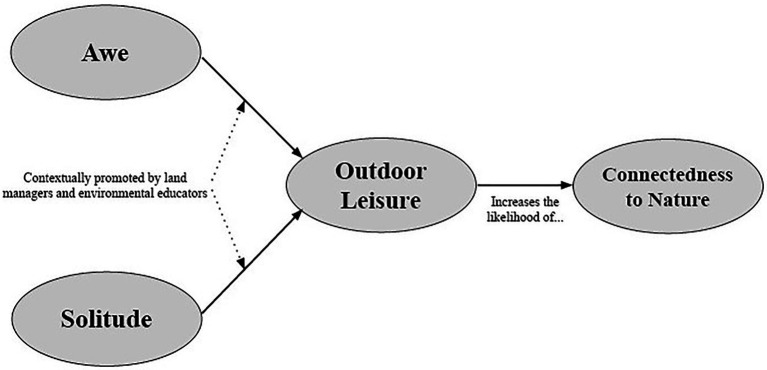
The proposed framework outlining the relationship between awe, solitude, outdoor leisure, and connectedness to nature in urban natural spaces.

Eudaimonic experiences facilitated by outdoor leisure may provide a pathway to this personal connection with the natural world. Awe and solitude, specifically as components of outdoor leisure, may provide the context for individuals to develop an ethical relationship with the natural environment. By challenging individuals’ frames of reference (Bai et al., 2017), awe inspired by the natural environment may encourage individuals to contemplate the broader workings of the world and ways they fit into these systems (Pieper, 1963). Additionally, solitude in the outdoors may allow individuals to escape from the frenetic nature of their daily lives and provide them space for contemplation, an important aspect of leisure (Pieper, 1963; Holba, 2013). Cumulatively, it is the integration of these elements that can provide a context for personal, eudaimonic experiences in relation to the natural environment, aligning with the assertion that leisure experiences are an end in and of themselves rather than a means to an end (Pieper, 1963). Similar spiritual and intrinsically motivated experiences with the outdoors have been articulated through the Norwegian concept of *friluftsliv* (e.g., Beery, 2013; Løvoll, 2019; Graves et al., 2020) These intimate experiences in the outdoors may allow individuals to develop the personal connection and care for the outdoors that Leopold (1949) argues must preclude development of healthy social-ecological systems. These intrinsically motivated and personal experiences in the outdoors may also potentially influence ways individuals view themselves in relation to the natural environment (Clayton, 2003). Viewing oneself as part of the natural environment, rather than separate from it (i.e., an environmental identity), generally links to a range of pro-environmental behaviors (Udall et al., 2020). If urban land managers and environmental educators can look to awe and solitude as contextual factors to promote outdoor leisure experiences, individuals may also be more likely to develop this personal identification with the natural environment. The framework outlined in this, and previous, sections is summarized in Figure 1.

## Promoting Awe and Solitude in Urban Outdoor Spaces

If awe and solitude provide contexts that promote outdoor leisure and eudaimonia, land managers and environmental educators may play a useful role in facilitating these experiences. As Cheesbrough et al. (2019) state, “Any particular landscape is not intrinsically health promoting, but rather the experience of the place produces effects that may be healing” (p: 43). Thus, land managers and environmental educators may act as catalysts for these healthy experiences. Following the arguments provided in the previous sections that outline ways awe and solitude may promote outdoor leisure experiences, literature associated with experiences of awe and solitude in urban outdoor spaces specifically is reviewed.

### Awe in Urban Outdoor Spaces

Cronon (1996) calls for seeing “wildness” embedded within our surroundings at all times, rather than seeing “wilderness” as a distant, otherized construct. This perspective asserts that awe, and the inner contemplation it may invoke, can be found in a wide variety of natural spaces with different levels of human presence (Cronon, 1996; Heintzman, 2009). The deconstruction of this binary between social and ecological systems has received widespread support in the academic literature (e.g., Haila, 2000; Oetelaar, 2014; Linnell et al., 2015). Despite this, the reviewed literature suggests that feelings of awe associated with large, rural natural areas (e.g., Loeffler, 2004; D'Amato and Krasny, 2011; Anderson et al., 2018) have been researched more frequently in comparison to urban outdoor areas. This may potentially limit how awe is understood in relation to the natural spaces within cities, also constraining our understanding of how leisure and eudaimonia can be promoted as well.

Despite this imbalance, several notable studies have examined awe in urban parks. Cheesbrough et al. (2019) utilized a photovoice methodology to explore how residents in Edmonton, Canada attached meaning to outdoor spaces throughout the city. Park visitors described feelings of awe in conjunction with feelings of spirituality and perspective on life when immersed in the natural environment (Cheesbrough et al., 2019). Moffat et al. (2009) provide a unique complement to this study through an ethnographic exploration of youth marijuana use in local natural areas and how this practice influences their connectedness to the natural world. While marijuana use has been considered a precursor to other unhealthy habits (e.g., Fergusson and Horwood, 2000), teenagers who smoked marijuana in local outdoor spaces cited the experience as being uniquely influential toward their sense of awe in relation to the natural world. These experiences were further described as a “gateway” to connectedness with the natural world (Moffat et al., 2009).

These qualitative findings are further corroborated by a small body of social psychological studies supporting the benefits of awe in urban natural settings. Broadly, general research in social psychology has linked awe to prosocial and pro-environmental sentiments (e.g., Piff et al., 2015; Zhao et al., 2018; Li et al., 2019). Specifically, regarding urban outdoor spaces, findings reported by Lopes et al. (2020) suggest that a walk as short as 30 min in an urban park can reduce feelings of rumination with awe acting as a mediator between experimental condition (walking in an urban park or along the street) and levels of rumination for one of the developed models (Lopes et al., 2020). Further, Collado and Manrique (2020) found that exposure to awe-evoking images, of both natural and built scenes, have positive cognitive effects for individuals. The positive influence across both image types (built and natural) may hold insight into how urban parks, given their embeddedness within cities, may invoke awe and its positive psychological outcomes for visitors.

While not explicitly examining feelings of awe, other research on urban outdoor spaces indicates park visitors may experience other outcomes related to awe such as spirituality (e.g., Krenichyn, 2006; Svendsen et al., 2016) and introspection (Shin et al., 2005). Furthermore, a recent literature review on positive mental outcomes associated with urban outdoor spaces builds upon this evidence. Pulling mostly on research outside of the urban context, the authors cite awe as a potential mechanism for nature to develop intrinsic motivation and self-discovery within urban park visitors (Leavell et al., 2019). Collectively, previous research suggests that experiences of awe in urban outdoor spaces closely aligns with the intrinsic, contemplative, and eudaimonic characteristics of leisure experiences (Holba, 2013; Dattilo and Lopez Frias, 2020). This information on awe in urban outdoor spaces provides direction for future research to expand upon this relatively small body of work while also providing useful guidance for practitioners in urban communities.

### Solitude in Urban Outdoor Spaces

Much research examining solitude in urban outdoor spaces discusses the construct in conjunction with other experiences such as “reprieve” or “escape” (e.g., Chiesura, 2004; Thompson et al., 2005). Being around non-human flora and fauna (Cheesbrough et al., 2019) and greater exposure to “natural” sounds in comparison to anthropogenic noise (Gidlöf-Gunnarsson and Öhrström, 2007; Tse et al., 2012) were often cited as two contextual factors promoting solitude in urban outdoor spaces. An open-ended survey of park visitors in Amsterdam, The Netherlands indicates that many individuals go to urban parks to remove themselves, both physically and mentally, from the stress associated with living near many people (Chiesura, 2004). Similar desires to seek solitude in urban outdoor spaces were expressed by residents in other cities such as Hong Kong, China (Wong and Domroes, 2004), New York City, United States (Svendsen et al., 2016), and Kuala Lumpur, Malaysia (Sreetheran, 2017). Solitude promoted by urban parks further relates to various health benefits such as providing space for contemplation (Kim et al., 2020), self-expression (Svendsen et al., 2016), and developing a closer relationship with the natural world (Cheesbrough et al., 2019).

It should also be noted that literature suggests that the desire or ability to experience solitude in urban outdoor spaces may not be culturally universal (e.g., Wesely and Gaarder, 2004; Jim and Chen, 2006; Wessels et al., 2021). For example, in a survey administered to visitors across urban parks in six cities throughout South Korea, solitude/privacy was reported as the least important outcome of 16 options provided (though solitude/privacy was still rated as “moderately important” or higher for residents across five of the six cities; Shin et al., 2005). Alternatively, in the Nelson Mandela Bay Municipality, South Africa, many individuals were hesitant to enter local parks alone due to safety concerns (Wessels et al., 2021). Depending on the broader cultural and social context within which urban outdoor spaces are embedded, solitude may not be a desired or feasible experience for some. Similar limiting factors may also exist for specific social groups in urban areas as well. Park characteristics, such as overgrown brush, may help some individuals feel a sense of solitude and escape from the built city environment (Cheesbrough et al., 2019). Alternatively, for others, the same overgrown brush may contribute to some individuals feeling unsafe due to factors such as decreased visibility (Kuo et al., 1998). Similar tensions may exist over law enforcement presence in urban parks (e.g., Slater et al., 2013; Mowatt, 2018). Reviewed literature suggests that the tension between facilitators and barriers toward solitude should be considered by land managers and environmental educators in urban outdoor spaces when aiming to facilitate leisure experiences.

## Recommendations for Facilitating Leisure in Urban Outdoor Spaces

Land managers and environmental educators may look to awe and solitude as contextual factors to support leisure in urban outdoor spaces, potentially resulting in greater connectedness to nature for visitors. Previous research has suggested that spatial availability of parks is not enough to encourage use; the characteristics of outdoor spaces also matter (Hughey et al., 2016; Rigolon, 2017). This must be acknowledged if investments in urban outdoor spaces are to be maximized. Somewhat unsurprisingly, the reviewed literature suggests that exposure to natural sights and sounds facilitates both awe and solitude for urban park visitors. While providing beneficial aspects to the visitor experience, the nature of these natural sights and sounds may influence the likelihood of individuals experiencing awe, solitude, and subsequently, leisure. While some individuals may experience awe and solitude readily in a woodland stewarded for its “natural” characteristics (Cheesbrough et al., 2019), others may feel unsafe in areas that are overgrown, unlit, or less intensively managed in general (Kuo et al., 1998). These divergent needs to experience awe and solitude may necessitate intentional managerial approaches in facilitating contexts to promote leisure. Utilizing strategies to satisfy various needs for leisure experience, such as the recreation opportunity spectrum (e.g., Xiao et al., 2018), may provide useful guidance for land managers and environmental educators. The recreation opportunity spectrum creates “zones” within an outdoor space where certain areas are managed to promote specific outdoor activities or experiences (Joyce and Sutton, 2009; Xiao et al., 2018). While traditionally utilized to meet the needs of various recreation activities with conflicting requirements in parks or protected areas, a similar approach may be helpful in providing contexts to facilitate awe, solitude, and leisure for visitors as well. Reviewed literature suggests that exposure to different types of flora and fauna (Kuo et al., 1998), soundscapes (Tse et al., 2012), as well as built and natural environments (Cheesbrough et al., 2019) may influence whether some individuals experience leisure in some settings and not others. Given the intrinsic nature of leisure (Pieper, 1963; Holba, 2013; Dattilo and Lopez Frias, 2020), individuals may gravitate toward the areas in park settings that satisfy these personal inclinations. Thus, adapting the recreation opportunity spectrum to facilitate contexts for awe and solitude may present a possible pathway to maximize investments in urban park management.

Additionally, the aggregated literature suggests that visitors to urban outdoor spaces experience awe and solitude in contexts that extend beyond what may be considered “traditional” outdoor experiences (e.g., hiking and biking; Outdoor Foundation, 2020). The reviewed literature outlines a variety of ways that park visitors found pathways to experiencing awe and solitude. The presented studies emphasize that unique individuals in unique contexts use urban parks in very different ways. While certain activities, such as walking and hiking, were referenced frequently (e.g., Krenichyn, 2006; Lopes et al., 2020), park visitors also found awe and solitude through less recognized activities like smoking marijuana (Moffat et al., 2009), artistic expression (Svendsen et al., 2016), and simply laying underneath trees (Burgess et al., 1988). While providing contexts to support some activities, like smoking marijuana, may be questionable (e.g., Fergusson and Horwood, 2000), land managers and environmental educators may be able to work more effectively with communities to meet diverse activity-based needs in order to facilitate leisure and eudaimonia. A process of co-creation regarding urban outdoor spaces may allow for community members to have a tangible voice in how investments in their local outdoor spaces are utilized, allowing them to advocate for their own ways of finding awe, solitude, and leisure. Practitioners and scholars may look to previous projects utilizing a transdisciplinary research lens for guidance on how to go about this (e.g., Mauser et al., 2013; Bergendahl et al., 2018). The transdisciplinary approach generally calls for a research process that is community-based and collaborative (Lang et al., 2012). While generally outlining how to go about research in a more practical and applied manner, a similar approach can be applied when designing urban park spaces, developing environmental education curriculum, and creating policies relevant to urban outdoor spaces. The transdisciplinary framework outlined by Lang et al. (2012) calls for regular discourse between stakeholders in what is called a “co-creative” process. This collaborative approach to promoting leisure in urban outdoor spaces may allow for communities to find leisure experiences and develop parks spaces that are uniquely meaningful to them.

## Conclusion

Distinguishing itself from outdoor recreation due to the intrinsic and contemplative aspects of the experience, outdoor leisure may serve as a pathway to connect individuals with the natural world in urban settings. Awe and solitude may serve as two contextual factors that promote this experience. To enhance the likelihood of this outcome, land managers and environmental educators may aim to find ways of stewarding outdoor areas and facilitating experiences that promote these elements of the park visitor experience. Reviewed literature suggests that embracing the embeddedness of urban parks within the city setting, managing for a range of environments to facilitate awe and solitude within urban parks, and understanding community-driven ideas of what it means to utilize urban parks in a meaningful way may all help to maximize the likelihood of outdoor leisure experiences for park visitors. To build resilient and thriving social-ecological systems within cities, outdoor leisure may represent and useful yet underutilized concept in building connectedness to nature.

## Author Contributions

TM is responsible for the conceptualization, literature review, and writing for this manuscript.

## Funding

Support for this research was provided by the Travel and Tourism Research Endowment in the College of Health and Human Development, The Pennsylvania State University; National Science Foundation DGE 2129893; and National Science Foundation DGE 1828822.

## Conflict of Interest

The author declares that the research was conducted in the absence of any commercial or financial relationships that could be construed as a potential conflict of interest.

## Publisher’s Note

All claims expressed in this article are solely those of the authors and do not necessarily represent those of their affiliated organizations, or those of the publisher, the editors and the reviewers. Any product that may be evaluated in this article, or claim that may be made by its manufacturer, is not guaranteed or endorsed by the publisher.
